# Supplementary material for: Causal interplay between lactose intolerance and gut microbiota: a combined bidirectional Mendelian randomization and in vivo validation study
Source: Front Nutr. 2026 Jun 1;13:1803337. doi: 10.3389/fnut.2026.1803337 (PMC13265576; doi:10.3389/fnut.2026.1803337)
Supplement: Supplementary file 1 [file Data_Sheet_1.zip › supplementary materials/Forward/leave one out cross validation/ebi-a-GCST90027458.finngen_R12_E4_LACTONAS.pdf]

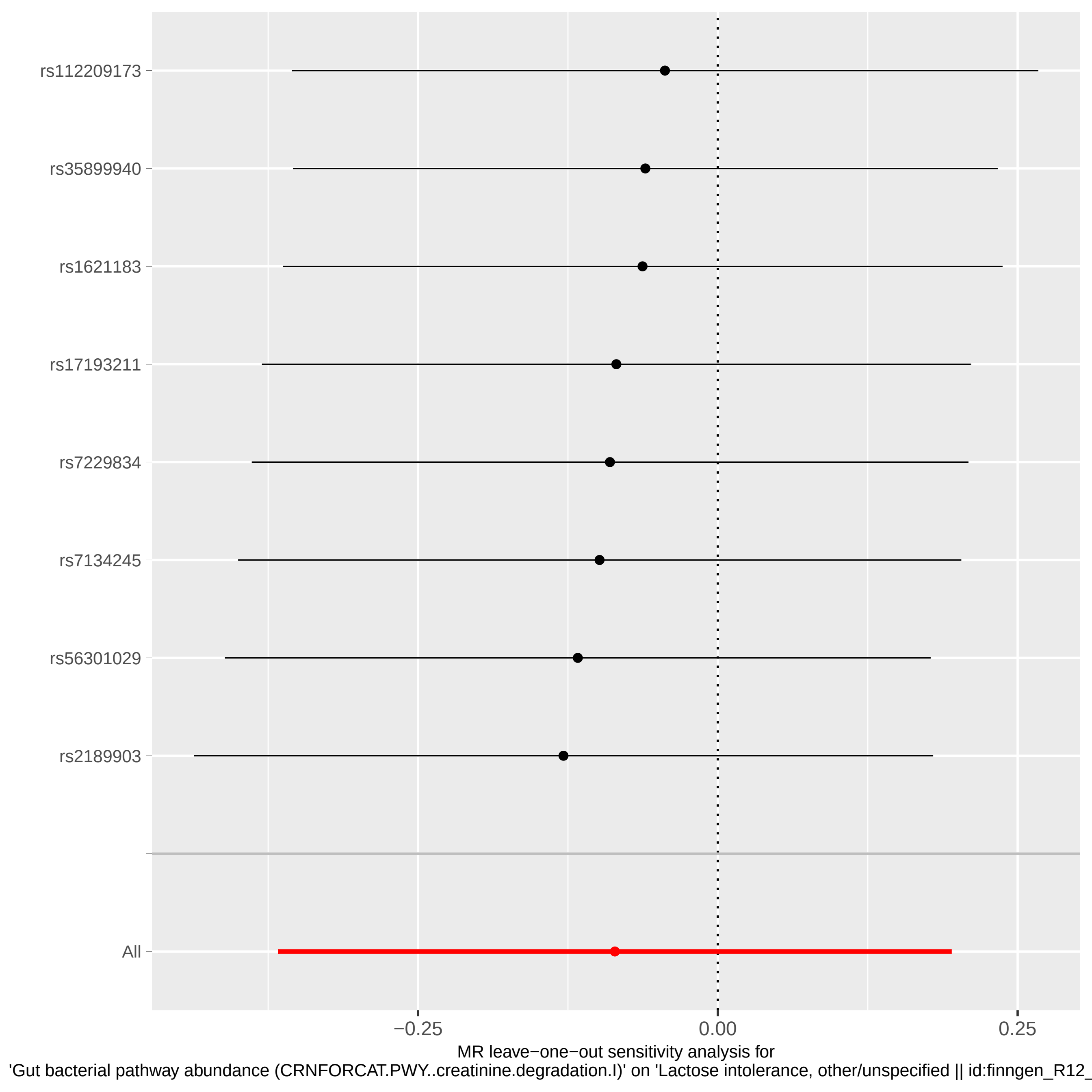
rs112209173
rs35899940
rs1621183
rs17193211
rs7229834
rs7134245
rs56301029
rs2189903
All
−0.25
0.00
0.25
MR leave−one−out sensitivity analysis for
'Gut bacterial pathway abundance (CRNFORCAT.PWY..creatinine.degradation.I)' on 'Lactose intolerance, other/unspecified || id:finngen_R12_
